# 自适应精准控温型液相色谱柱温箱的设计与评价

**DOI:** 10.3724/SP.J.1123.2023.11021

**Published:** 2025-02-08

**Authors:** Xingfa REN, Fengwei SHAO, Yong WU, Xinming LIAO, Zhuo WANG, Linjuan ZHOU, Xuhong HE, Weibing ZHANG

**Affiliations:** 1.华东理工大学,上海200237; 1. East China University of Science and Technology, Shanghai 200237, China; 2.月旭科技(上海)股份有限公司,上海201613; 2. Welch Technology (Shanghai) Co. Ltd., Shanghai 201613, China; 3.浙江月旭材料科技有限公司,浙江金华321000; 3. Welch Materials (Zhejiang), Inc., Jinhua 321000, China

**Keywords:** 精准控温, 算法, 传热原理, 液相色谱, 柱温箱, precise temperature control, algorithm, heat transfer principle, liquid chromatography, column oven

## Abstract

高效液相色谱系统作为诸多领域的关键分析手段,提升其分离效率、稳定性和普适性是研究重点。在该技术手段中,流动相的组成和比例、固定相的种类以及色谱柱的尺寸等显著影响分离效果,色谱柱和流动相的温度对分离效果也起到了至关重要的作用。高效液相色谱系统通常采用柱温箱进行温度控制,高精度的温度控制可提升色谱分离性能、缩短分析时间、确保分析结果的重复性。本文创新性地改进了柱温箱的结构,通过融合多种先进温度控制算法,实现了高精度、宽范围(4~90 ℃)的连续升降温控制,为色谱分析方法发展开辟了新路径。此改进摒弃了复杂的硬件系统,仅通过软件优化即达成了高度稳定的控温效果,控温精度提升至±0.1 ℃。此外,通过优化保温结构与采用环保隔热材料,也增强了控温精细化能力;对于热源热传导机制的研究,进一步提升了柱温箱的综合性能。结果表明,该改进显著提高了色谱分离的重复性和稳定性,为高效色谱分析方法的发展奠定了坚实基础。

## Abstract (en)

High performance liquid chromatography (HPLC) is a key analytical technique that is used in a number of fields. Improving the separation efficiency, stability, and universality of HPLC has been a continuing analytical-chemistry focus. In chromatographic separation, factors such as the composition and ratio of the mobile phase, the type of stationary phase, and the dimensions of the chromatographic column significantly affect the separation efficiency. In addition, the temperatures of the chromatographic column and mobile phase are also important for achieving separation. The column oven is usually used to stably control the column temperature in the HPLC separation system. Indeed, highly accurate temperature control ensures superior separation performance, short analysis times, and repeatability. In this study, we innovatively improved the traditional column oven by combining a variety of temperature-control algorithms to deliver continuous and highly accurate temperature control in the wide 4-90 ℃ range, and by exploring a new chromatographic-method development route. Instead of focusing on the complex hardware system, we optimized the software to achieve highly stable and accurate (±0.1 ℃) temperature control. Temperature-control performance was further improved by optimizing the structure of the thermal insulation and employing reliable and environmentally friendly thermal-insulation materials. Additionally, the thermal conduction of the heat-source device is discussed based on the heat-transfer principle with the aim of improving the performance of the column oven. The improved column oven delivered significantly enhanced chromatographic-separation repeatability and stability thereby reliably guaranteeing the development of highly efficient chromatographic analysis methods.

为了提高液相色谱分离的选择性和效率,研究人员将注意力主要集中于调节流动相的组成和比例、选择固定相的种类和尺寸以及选择色谱柱的尺寸等^[1]^。近年来,越来越多的研究人员开始关注温度对液相色谱分离的影响,并提出了一系列相应的理论^[2,3]^。柱温箱作为高效液相色谱系统的重要组件,不同的生产厂家所采用的控温方法有很大不同。在设定温度下,精准的温控系统可以避免柱温受外界的影响,从而保持柱温恒定,对保证分析结果的重复性和准确性有非常重要的作用。

通过理论和实践的相互印证,针对大多数样品的分析方法发展而言,适当升高温度通常更有利于改善分离效果^[4⇓⇓⇓⇓⇓-10]^。温度对液相色谱分离的影响可以从热力学和动力学两方面体现。从热力学角度讲^[11]^,依式(1)范特霍夫方程中温度(*T*)与保留因子(*k*)之间的关系:


(1) lnk=−ΔH/RT+ΔS/R+lnβ

其中*R*为气体常数,*β*为相比,Δ*H*和Δ*S*分别表示溶质从流动相转移到固定相时的焓差和熵差。

温度升高,化合物的*k*值减小,有利于快速分析。对于不同种类的化合物,其在流动相和固定相之间交换的熵和焓不同,因此可以通过改变柱温改善分离选择性。温度对分离选择性的影响在手性分离中尤为突出,Maisuradze等系统研究了不同温度下酮洛芬对映体在涂覆型和键合型多糖类手性柱上的分离情况,也说明了熵焓补偿的特征^[12⇓⇓-15]^。从动力学角度讲,柱温不仅影响样品沿纵向和径向的扩散,也直接影响溶质在两相间的传质速率,其综合影响依不同的分离模式和样品结构呈现出分离选择性与柱温不同的依赖关系。此外,柱温升高,流动相黏度变小,分析速度可显著提高。

通常的液相色谱柱温箱仅具有加热功能,一般控温范围为(室温+5)~80 ℃,不能满足复杂分离体系的实际要求。柱温箱大多采用风浴控温,以适用升温、降温、室温保持、极端变温、低温等多种应用场景。为满足色谱分析对柱温箱控温精度和控温范围的更高要求,研究者通过制定出合理、高效的控温策略,开发灵敏度高、抗干扰性强的控制电路,设计灵活多变的控温算法,实现柱温箱的精准升温、降温功能。不同柱温箱也都基于其仪器结构采用相应的设计理念及控温策略以实现更好的功能。文献[16,17]采用模糊控温策略、比例(proportional,P)-积分(integral,I)-微分(derivative,D)控制(PID)或模糊PID控温策略,能够实现一般控温的要求,但是要进一步达到更快速、更高精度的控温要求,还需要优化出更有效的算法。文献[18]采用粒子群算法实现PID参数自适应,有效提高了控制精度;文献[19]基于反向传播(BP)神经网络控制算法对PID控制参数进行优化,提升了控制效果,然而粒子群算法和BP神经网络控制算法仅限于仿真实验,其运算量较大,难以在运算力有限的实际嵌入式系统中实现。

为此,本工作设计了一种自适应精准控温型液相色谱柱温箱。采用半导体制冷片,一面贴附在恒温箱内导热器件上,另一面贴附在恒温箱外散热器上,通过给制冷片通入电流,能够对恒温箱进行升降温操作,将恒温箱的温度控制在一个精确的范围内。结合多次曲线回归校准算法、双PID算法^[20]^、极端脉冲宽度调制(pulse width modulation,PWM)逼近算法、卡尔曼预测算法和双珀尔帖跟随算法等核心算法,可针对不同的应用场景,自动判断并采用一种或多种算法程序进行控温,从而为最优分析条件的选择提供保障。

## 1 自适应精准控温柱温箱的设计

### 1.1 结构布局

采用立式设计布局,如图1a所示,最多可同时放置6根300 mm长的分析型色谱柱,内腔空间约为12 L,采用两片高功率珀尔帖模块进行加热或制冷,珀尔帖器件表面的制热量或制冷量通过铝件传导,经耐高温风扇导入腔体内,通过微循环风道形成循环风。这样的设计不仅能充分利用实验室的垂直空间,节约台面空间的占用,也更有利于保温、热传导结构和微循环风道的设计,使得热交换的时间较卧式布局更短,更小的热交换延迟提升了热传导效率;图1b显示了对柱温箱腔体内部的热流体分析,展示了热量的传递轨迹和分布,从而保证了柱温箱的控温精度。

**图 1:**
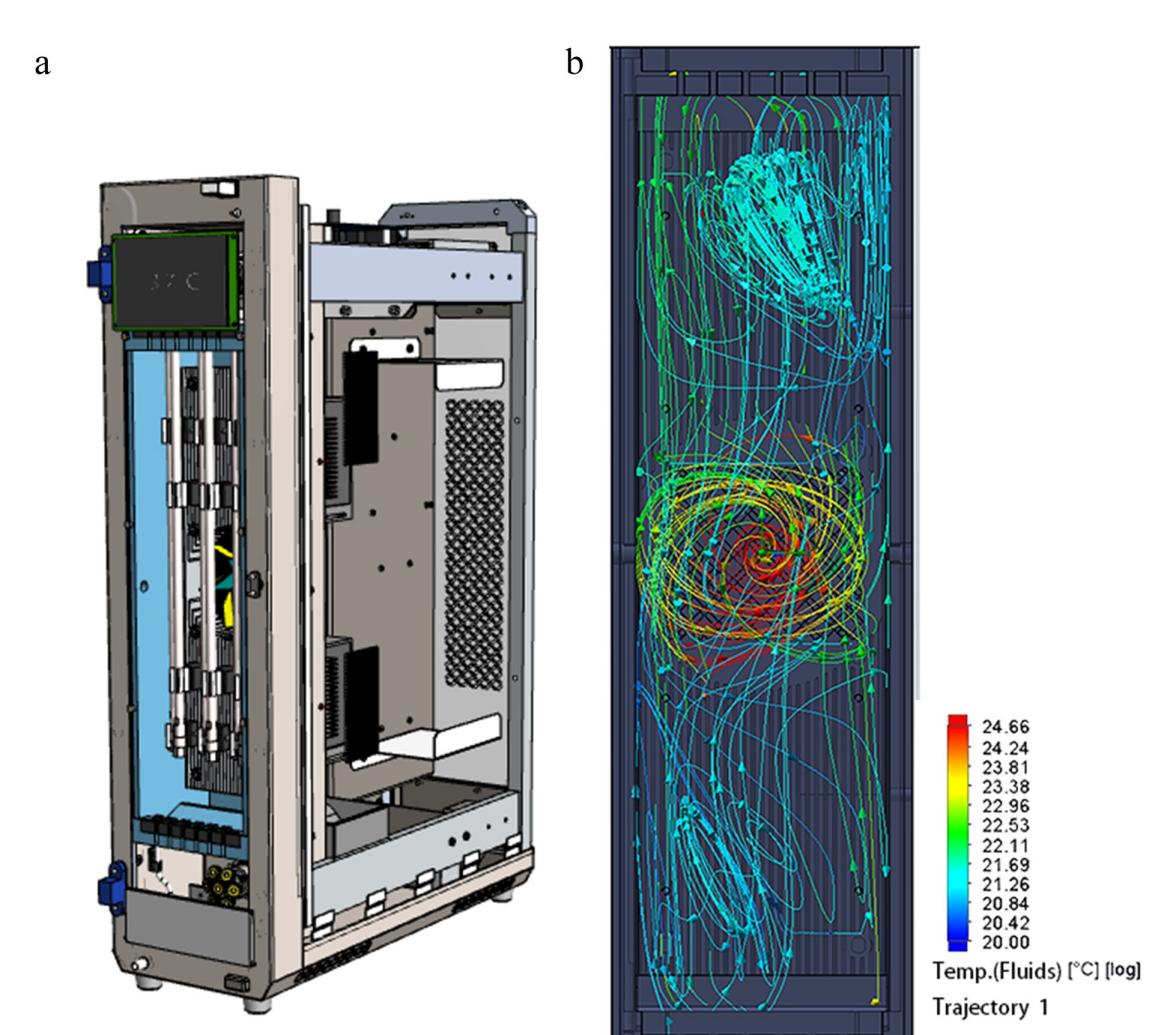
(a)柱温箱结构设计图及(b)柱温箱内循环流体模拟图

### 1.2 控制系统

柱温箱的控制系统示意图见图2。

**图 2:**
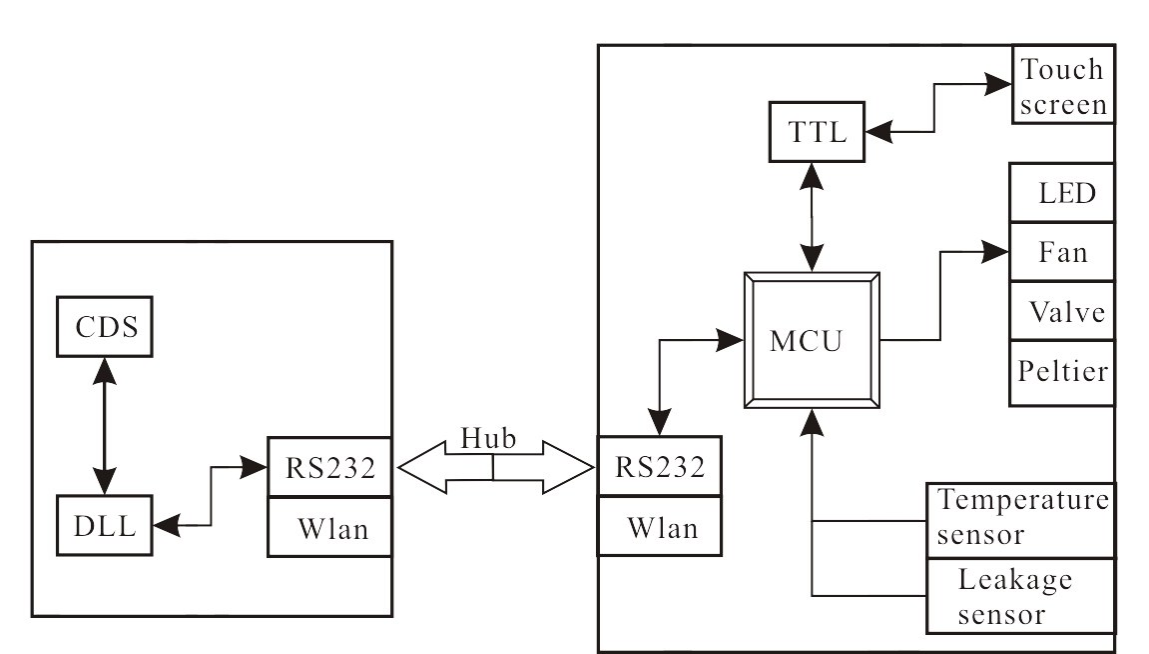
柱温箱控制系统示意图

柱温箱温度的精确控制系统由两部分组成,一部分为上位机数据管理系统(CDS),利用仪器动态链接库DLL指令通过RS232或Wlan网口与柱温箱连接,实现对柱温箱的控制和信息反馈显示;另一部分为柱温箱本地控制系统,主要由一颗32位微处理器MCU、4路高精度温度传感器(Temperature sensor)、一个漏液检测传感器(Leakage sensor)、工作状态指示灯(LED)、柱温箱循环风扇(Fan)、柱切换阀(Valve)组成。MCU根据CDS或触摸屏(Touch screen)给出的目标温度指令,实时监测柱温箱内Temperature sensor和室内Temperature sensor反馈的温度,计算出最优的控制算法。根据算法,驱动珀尔帖(Peltier)对柱温箱内部进行加热或制冷。为了实现高精度的控温,4路温度传感器(1路为柱温箱内部温度,2路为珀尔帖表面温度,1路为室温)均采用了高精度的1/3B级PT1000温度传感器,可以将极其细微的温度变化检测出来;通过24位A/D转换芯片及高精度的参比电阻组成的采样电路,实时采集温度的变化,通过内置控制程序实时调控,保证了柱温箱腔体内部的温度波动最小。

## 2 控温策略、算法及实际应用

### 2.1 控温策略

针对不同应用场景,我们对柱温箱整体控温策略进行了分别设计(见表1)。基于南北方实验室室温、早晚和四季温度的变化对柱温箱控温产生的微小影响,结合表1中的控温策略,开发了相对完善的控温算法模型,包括多次曲线回归校准算法、双PID算法、极端PWM逼近算法、卡尔曼预测算法和双珀尔帖跟随算法,仪器可根据应用场景的变化自动组合出最优的控温策略进行控温。

**表1:** 柱温箱控温策略

Application scenario	Room temperature condition	Temperature difference	Instruction
Target temperature>cavity temperature	relatively stable	large	fast heating➝slow heating
	relatively stable	small	slow heating
	slowly rising	large	slow heating
	slowly declining	small	slow heating
Target temperature<cavity temperature	relatively stable	large	fast cooling➝slow cooling
	relatively stable	small	slow cooling
	slowly rising	large	fast cooling→slow cooling
	slowly declining	little	slow cooling
Target temperature≈cavity temperature	relatively stable	minimal	quick switching between
≈room temperature			heating and cooling

### 2.2 算法介绍

多次曲线回归校准算法:主要用于进一步消除温度信号传递过程产生的误差。通过多点曲线回归算法,使各个温度点与标准温度计的读数一致。

双PID算法:即2个PID算法,分别控制加热和制冷,因为加热和制冷过程不一样,加热是得到热量,制冷是失去热量,产生了加热、制冷2组独立的PID控制算法;系统通过对当下的环境温度与设定温度进行比较,判断是要加热还是制冷,然后启用相应的PID算法进行精确的温度控制。

极端PWM逼近算法:在设定温度与环境温度极为接近的情况下,PID算法计算的增益无法满足精确控温要求,此时系统会自动切换至极端PWM逼近算法进行微调,以确保温度保持稳定。

卡尔曼预测算法:根据先前的温度变化预测未来的温度变化,在接近设定目标温度时进行提前干预,减少不必要的温度过冲。

双珀尔帖跟随算法:为了满足特定的升温速率要求并确保高温度均匀度,我们的柱温箱采用了两块珀尔帖元件进行加热或制冷。该算法可使这两块珀尔帖以主、从模式协同工作,其中主珀尔帖的状态用于调控从珀尔帖的PWM功率输出。这一策略有效消除了因两块珀尔帖性能差异而引起的功率不平衡问题。

### 2.3 精确控温实现方式

控温方式主要包括4种:正常控温、PID控温、微调控温以及保持控温。根据目标温度与环境温度的差别,自适应选择不同的控温方式(见表2)。该控温策略可以使柱温箱有效地适用于南、北方环境温差及夏季、冬季温差悬殊的环境。

**表2:** 精确控温实现方式

Condition	Operation	Algorithms
A<B	normal heating	heating PID algorithm and dual Peltier following algorithm
A≥B≥D	finely tuned heating	extreme PWM algorithm
B<C	normal cooling	cooling PID algorithm and dual Peltier following algorithm
C≤B≤D	finely tuned cooling	extreme PWM algorithm

A: room temperature+5 ℃; B: target temperature; C: room temperature-5 ℃; D: room temperature. PID: proportional integral derivative; PWM: pulse width modulation.

### 2.4 控温策略在精确控温中的应用

#### 2.4.1 升温测试

如图3所示,设定目标温度(*T*_Target_)为90 ℃,程序检测当前环境温度(*T*_Room_),若*T*_Target_> *T*_Room_+5 ℃,则执行正常的加热程序。

**图 3:**
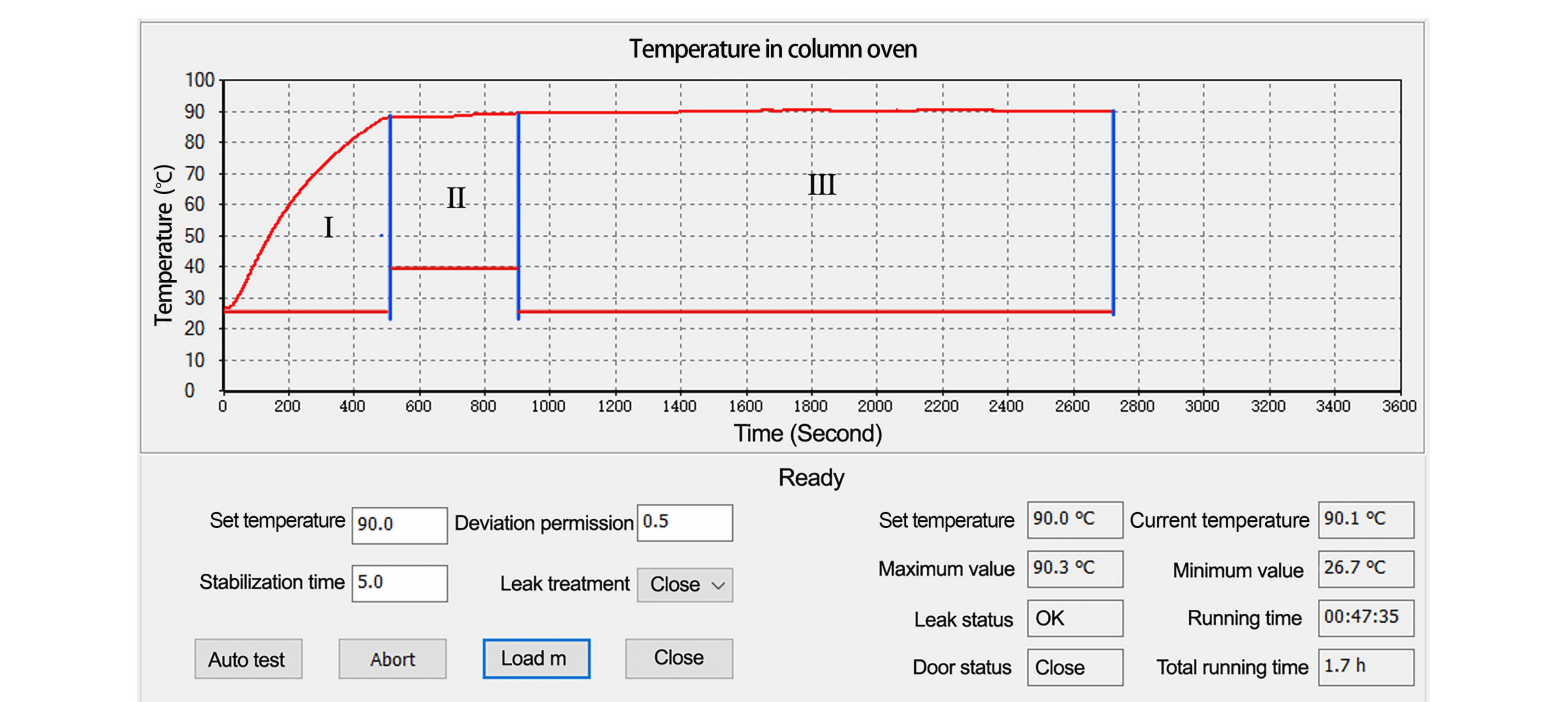
升温测试温度监测走势图

第一阶段:执行加热操作。此时以主珀尔帖模块温度作为控制目标,运行双珀尔帖跟随算法。将珀尔帖模块温度以最快的速度升到由卡尔曼算法预测的目标温度,每次运行后会计算得出一个温度数据并保存在数据寄存器中,作为下一次珀尔帖模块温度控制预测的目标温度。降低输出功率以维持珀尔帖模块的当前温度,防止温度过冲,减少温度的调节时间。

第二阶段:此时切换为以柱温箱内的温度作为控制目标,当实际柱温箱温度与*T*_Target_的差值<3.5 ℃时PID加热算法介入控制。调整珀尔帖模块的输出功率,使柱温箱内的温度无限接近设定的目标温度。PID算法具有自学习功能,在温度调节过程中会不断地优化P、I、D参数,并将其存储在这个温度区域的存储器中,用于下次控温时直接调用,从而提高控温精度,节省控温时间。

第三阶段:将卡尔曼算法与PID算法相结合,根据温度的变化不断预测接下来的温度趋势,使柱温箱在当前温度下产生的热量损失与从珀尔帖得到的热量相一致,保证其温度稳定。此时程序进入保持阶段,只要这个平衡不被打破,温度波动范围就可以控制在目标温度的±0.1 ℃以内。如果因外界因素打破这种平衡,程序会重新进行一次控温调节过程,使温度重新回到稳定状态。

#### 2.4.2 降温测试

与升温过程相仿,如图4所示,设定目标温度*T*_Target_为4 ℃,程序检测当前环境温度*T*_Room_,若*T*_Target_<*T*_Room_-5 ℃,则执行正常的降温程序。

**图 4:**
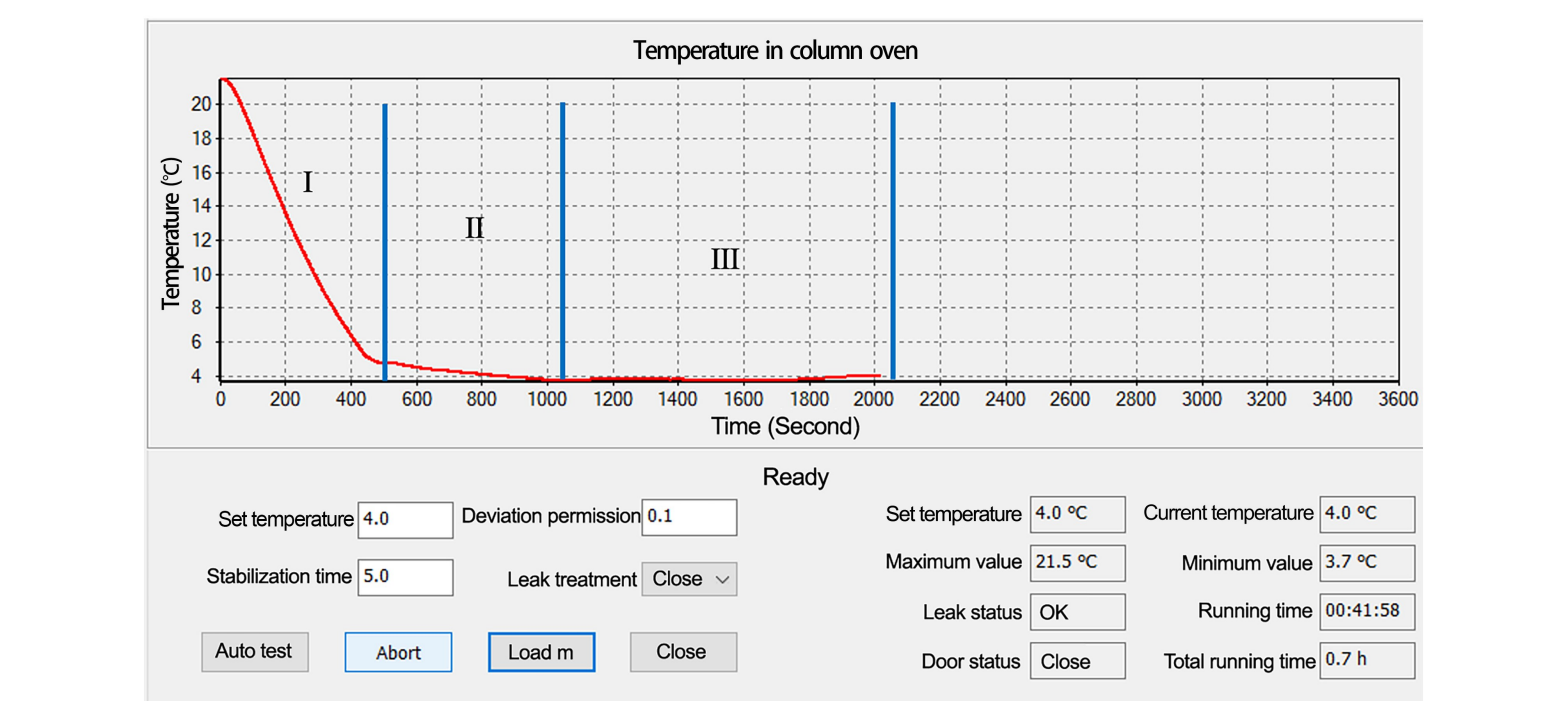
降温测试温度监测走势图

执行正常降温操作,首先将珀尔帖模块温度以最快的速度降到由卡尔曼算法预测的目标温度。其次系统自动切换为以柱温箱内的温度作为控制目标,差值<3.5 ℃时PID制冷算法介入控制,调整珀尔帖的输出功率,使柱温箱内的温度无限接近设定的目标温度。最后通过卡尔曼算法与PID算法相结合,控制目标温度值波动在±0.1 ℃内,实现温度的稳定控制。

## 3 柱温箱的性能评价

### 3.1 控温性能评价

在柱温箱腔体内的上下两个位置分别放置一个温度探头,分别从室温开始进行升温和降温试验,图5为柱温箱从室温25 ℃升温至35、50、60、70、80、90 ℃和从室温25 ℃降温至20、10、4 ℃的温度-时间曲线图。

**图 5:**
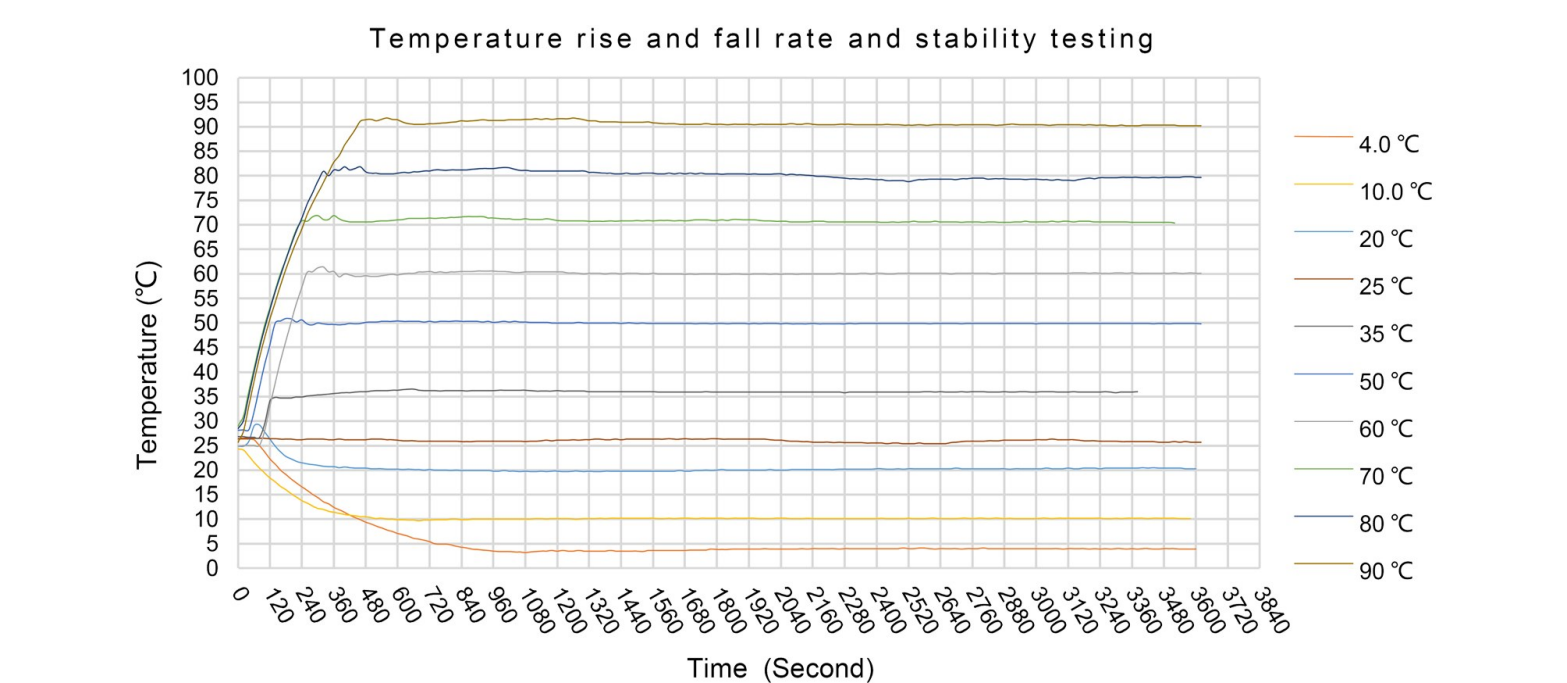
柱温箱从室温升、降至设定温度的测试图

从图5可以看出,除4 ℃温度点需要约1200 s进入预定温度范围并保持稳定,其他温度点均在700 s以内就可以进入预定温度范围并保持稳定。

实验结果表明,所有设定温度均可在1500 s内进入稳定保温阶段。

在柱温箱内布置8个测温点(CH1~CH8),如图6所示。对柱温箱进行极端升降温试验,以测试其性能。首先将柱温箱的温度从室温降至4 ℃,稳定后再从4 ℃升温至90 ℃,平衡稳定。这一过程所需要的时间约1400 s。进一步进行降温试验,将柱温箱温度从90 ℃降温至4 ℃,这一过程与升温类似,同样需要约1300 s的时间。整个测试过程中不打开柱温箱门,保证系统的稳定性。

**图 6:**
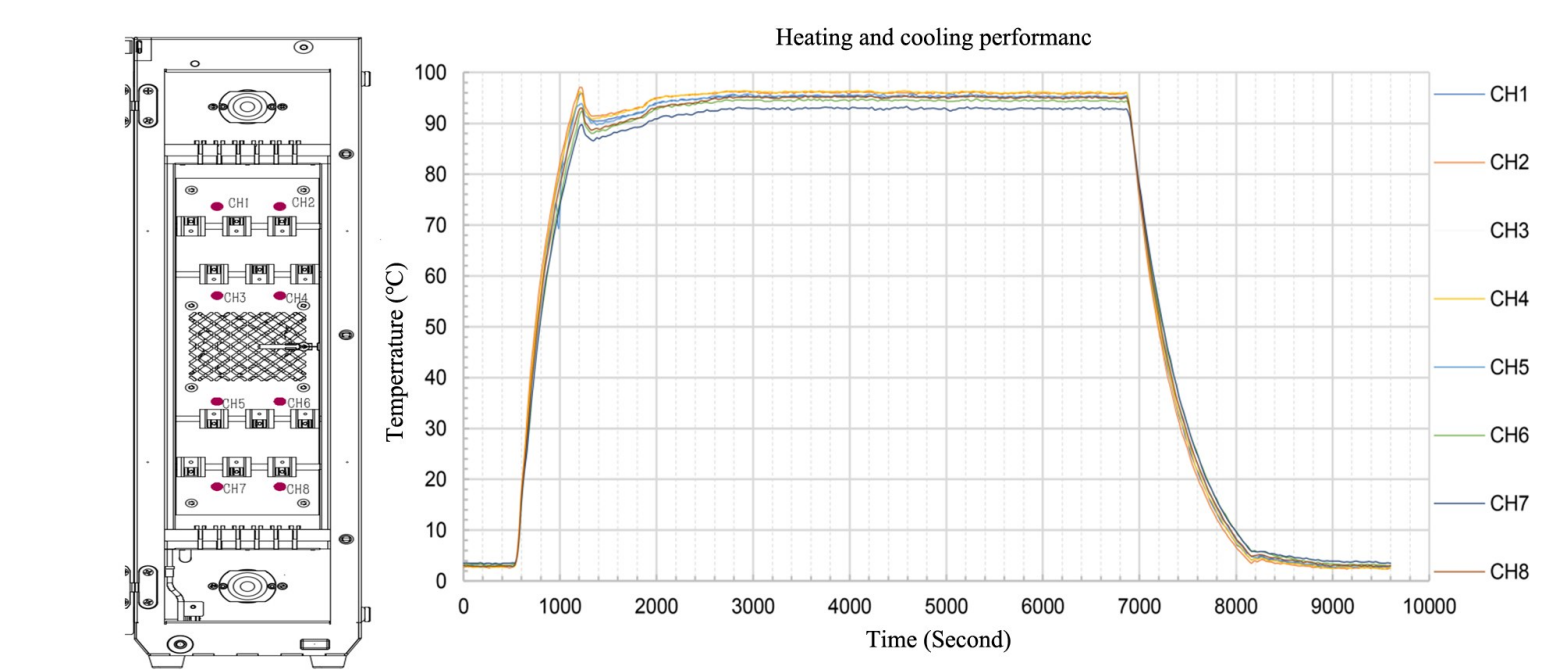
柱温箱极端升降温测试图

图6的结果也表明,柱温箱的升降温速度相当,能够进行快速温度切换。这一性能不仅可以为HPLC方法发展提供恒温操作、程序升温操作,也能够提供程序降温操作,甚至升降结合变温操作,使色谱方法的发展更加灵活。

### 3.2 控温准确度和稳定性评价

参照GB/T 26792-2019《高效液相色谱仪》^[21]^的4.4节恒温箱温度设定值误差和控温稳定性的测试方法,对柱温箱的4、10、20、25、35、50、60、70、80和90 ℃控温测试点进行温度准确度和稳定性评价。当温度显示值稳定后,每隔10 min记录一次温度计显示温度,共7次,求出平均值。平均值与设定值之差为该温度下的设定值误差,作为该温度下的控温准确度;7次读数中的最大值与最小值之差为该温度下的控温稳定性误差,作为该温度下的控温稳定性。

计算方法:


(2) $$T=\bar{T}-T_{0}$$

(3) $$T_{c}=T_{\max}-T_{\min}$$

其中,Δ*T*为温度准确度, $\overset{-}{T}$ 为7次测量的平均值,*T*_0_为温度设定值,*T*_c_为控温稳定性,*T*_max_为7次测量最大值,*T*_min_为7次测量最小值,单位均为摄氏度(℃)。

在进行试验之前,对柱温箱内的3个温度传感器PT1000进行了温度校准,测试时设置其温度波动可接受限度为±0.1 ℃,并保持测试环境温度波动在±1 ℃/6 h以内。测试结果如表3所示,所有测试点温度在达到微调临界值后都能在500 s以内实现稳定,控温准确度在±0.1 ℃以内,稳定性可控制在±0.1 ℃以内。

**表3:** 主要控温点的控温准确度和稳定性

Temp./℃ ^1)^	Time/s ^2)^	Accuracy/℃	Stability/℃
4	480	±0.1	±0.1
10	300	±0.1	±0.1
20	240	±0.1	±0.1
25	500	±0.1	±0.1
35	480	±0.1	±0.05
50	240	±0.1	±0.1
60	300	±0.1	±0.1
70	240	±0.1	±0.1
80	280	±0.1	±0.1
90	300	±0.1	±0.1

1) temperature control point; 2) stabilization time required to reach the temperature-control point.

## 4 结论与展望

HPLC仪器系统中,柱温的控制精度直接影响所发展分析方法的稳定性和普适性。本文设计了一种新型自适应精准控温型液相色谱柱温箱,通过引入多种智能控温算法建立控温模型,结合设计精巧的风道系统和热传导结构,确保了柱温箱热交换的高效实现和温度精准控制。该柱温箱单点控温准确度可达到±0.1 ℃,稳定性可达到±0.1 ℃,能够在4~90 ℃的温度范围内精确控温;升降温效果相当,能够进行快速温度切换,使得色谱方法发展中温度调节更加灵活。新型高精度柱温箱对柱温的精准控制为进一步研究温度对色谱分离的影响提供了有力的支持和保障。研究色谱热力学和动力学中温度和柱效、分离度以及保留时间之间的关系,对于保证样品分析结果的重复性和稳定性皆具有积极意义。柱温的精准控制不仅可以极大地拓宽HPLC在药物的定性定量分析、石油生产鉴定、蛋白组学研究、有机污染物分离分析等领域中的应用,而且对于目前精准医学研究中涉及的温度敏感性分析方法的发展、生物分子之间相互作用研究等具有不可替代的作用。将多种算法结合进行控温的研究方法也为色谱泵的精准流量控制和压力的精确控制等提供了一套可行的方案,为HPLC系统综合性能的大幅度提升开拓了一条新思路。
